# A Systematic Review and Meta-Analysis: Does Molecular Hydrogen Exert Radioprotective Effects Without Compromising the Antitumor Efficacy of Radiotherapy or Concurrent Chemoradiotherapy?

**DOI:** 10.7759/cureus.116422

**Published:** 2026-09-17

**Authors:** Yu Ivy Zhang, Michiko Miyakawa, Ayako Miyakawa

**Affiliations:** 1 Department of Medical Biochemistry and Biophysics, Karolinska Institutet, Stockholm, SWE; 2 Faculty of Sustainability Studies, Hosei University, Tokyo, JPN; 3 Division of Urology, Karolinska University Hospital, Solna, SWE

**Keywords:** cancer, concurrent chemoradiotherapy, molecular hydrogen, radiation-induced toxicity, radioprotection, radiotherapy, tumour response

## Abstract

Radiotherapy and concurrent chemoradiotherapy (CCRT) are widely used for cancer treatment but are frequently associated with radiation-induced toxicities. Molecular hydrogen has been proposed as a potential radioprotective agent because it protects normal tissues from oxidative damage. However, an important concern is whether molecular hydrogen also protects tumor tissues.

This systematic review and meta-analysis aimed to evaluate whether molecular hydrogen preserves tumor response while providing radioprotection in cancer patients receiving radiotherapy or CCRT. The Preferred Reporting Items for Systematic Reviews and Meta-Analyses (PRISMA) guidelines were followed for conducting this review. Eligible studies were English-language original clinical studies of adult cancer patients (≥18 years) treated with radiotherapy or CCRT and receiving molecular hydrogen for radioprotection. Three electronic databases (PubMed, Embase, and Web of Science) were searched from inception to 8 July 2026. Seven studies involving 222 patients were qualitatively synthesized, of which 3 involving 130 patients were included in the meta-analysis. The random-effects model was used. The risk of bias was evaluated with validated tools. The risk ratio (RR) was used to estimate the pooled tumor response.

Molecular hydrogen did not significantly affect tumor response compared with the control group (RR = 0.98, 95% CI 0.83-1.15). Sensitivity analysis restricted to randomized controlled trials yielded a broadly consistent result (RR = 0.99, 95% CI: 0.84-1.17). The narrative synthesis further indicated that molecular hydrogen reduced radiation-induced toxicities, improved quality of life, and did not adversely affect hematological parameters. In conclusion, this systematic review and meta-analysis suggest that molecular hydrogen may have radioprotective effects in cancer patients receiving radiotherapy or concurrent chemoradiotherapy, with the currently available evidence showing no significant reduction in tumor response.

This review was registered with INPLASY (INPLASY202680108).

## Introduction and background

Approximately half of cancer patients will receive radiotherapy during treatment [1]. Despite substantial advances in radiotherapy techniques that have reduced radiation-induced toxicities, minimizing treatment-related adverse effects remains an important clinical challenge [2]. Common radiation-induced toxicities include dermatitis, mucositis, pericarditis, esophagitis, enteritis, nausea, fibrosis, fatigue, and hair loss. In addition, when radiotherapy is combined with cisplatin, patients may develop treatment-related hearing loss [3]. Concurrent chemoradiotherapy (CCRT), defined as the simultaneous administration of chemotherapy and radiotherapy, has become an established treatment approach for certain types of cancer [4].

Radiation exerts its damaging effects at the cellular level through both direct and indirect mechanisms [5]. Direct damage occurs when ionizing radiation interacts directly with biological macromolecules. Because biological macromolecules, particularly DNA, occupy only a small proportion of the total cellular volume, the probability of direct damage is relatively low. In contrast, indirect damage primarily results from the radiolysis of water [5-8]. Water radiolysis generates different types of reactive oxygen species (ROS), among which the hydroxyl radical (•OH) is highly reactive [9]. •OH is considered the major contributor to ionizing radiation-induced cellular injury [10]. It damages DNA by oxidizing deoxyguanosine to 8-hydroxy-2′-deoxyguanosine (8-OHdG) [11], induces lipid peroxidation that disrupts membrane integrity and permeability [12,13], and alters protein structure and function [14].

Molecular hydrogen (H₂) has emerged as a promising radioprotective agent. As the smallest gas molecule, H₂ diffuses rapidly across biological membranes and readily penetrates cells to exert its biological effects [15]. Based on a wide range of studies, including those beyond the field of oncology, the radioprotective effects of H₂ have been attributed to several mechanisms. H₂ selectively reduces and eliminates the highly cytotoxic •OH and peroxynitrite (ONOO-) [16,17]. Beyond its antioxidant properties, H₂ also suppresses oxidative stress-induced inflammation. H₂ causes a decrease in pro-inflammatory cytokines and an increase in anti-inflammatory cytokines [18,19]. H₂ also attenuates oxidative stress-induced cell death by preserving cell membrane integrity and maintaining mitochondrial function [20]. Its radioprotective effects have also been attributed to increased expression of endogenous antioxidant-related proteins [21].

An ideal radioprotector should reduce ionizing radiation-induced injury to normal tissues without compromising the antitumor efficacy of radiotherapy or CCRT [22]. Although H₂ scavenges •OH in normal tissues, an important concern is whether it exerts the same effect in tumor tissues, potentially reducing oxidative damage to tumors. Previous reviews have suggested that molecular hydrogen may provide radioprotection without attenuating tumor response. However, these conclusions have been based primarily on descriptive summaries of a limited number of clinical studies rather than on a quantitative synthesis and analysis of the available evidence [6-8]. Therefore, we aimed to quantitatively evaluate whether molecular hydrogen influences tumor response in cancer patients receiving radiotherapy or CCRT in this systematic review and meta-analysis.

## Review

Method

Reporting Guidelines and Protocol Registration

The Preferred Reporting Items for Systematic Reviews and Meta-Analyses (PRISMA) 2020 statement guided the conduct and reporting of this work [23]. The study protocol was registered with INPLASY (registration number INPLASY202680108).

Search Strategy

A comprehensive literature search was conducted in three electronic databases (PubMed, Embase, and Web of Science) from database inception to July 8, 2026. Search strategies combined free-text terms related to molecular hydrogen, radiotherapy, and cancer (Table 1). Searches were restricted to English-language publications, and filters for human studies were applied where available. Additional eligible studies were identified through manual examination of the reference lists of relevant reviews.

**Table 1:** Search strategies

Search Strategies	
PubMed	(hydrogen[tiab] OR H2[tiab]) AND (radiation[tiab] OR radiotherap*[tiab] OR irradiat*[tiab] OR radioprotect*[tiab]) AND (cancer*[tiab] OR tumor*[tiab] OR tumour*[tiab] OR neoplasm*[tiab] OR malignan*[tiab] OR oncolog*[tiab]); Filters: English, Humans
Embase	(hydrogen:ti,ab OR H2:ti,ab) AND (radiation:ti,ab OR radiotherap*:ti,ab OR irradiat*:ti,ab OR radioprotect*:ti,ab) AND (cancer*:ti,ab OR tumor*:ti,ab OR tumour*:ti,ab OR neoplasm*:ti,ab OR malignan*:ti,ab OR oncolog*:ti,ab); Filters: English, Human
Web of Science	TS=(hydrogen OR H2) AND TS=(radiation OR radiotherap* OR irradiat* OR radioprotect*) AND TS=(cancer* OR tumor* OR tumour* OR neoplasm* OR malignan* OR oncolog*); Filter: English

*Eligibility Criteria* 

The inclusion criteria were as follows: (1) Population: adult cancer patients (≥18 years) who received radiotherapy or CCRT as treatment; (2) Intervention: molecular hydrogen administered for radioprotection, irrespective of the mode of administration; (3) Outcomes: provided data on at least one clinically relevant outcome, such as tumor response, radiation-induced toxicities, patient quality of life, or other treatment-related outcomes; and (4) Study design: original clinical research published in English. The presence of a comparator group was not an eligibility criterion for study inclusion. However, only studies with a comparator group that did not receive molecular hydrogen were eligible for inclusion in the meta-analysis.

Studies were excluded if they fell into any of the following categories: non-human studies, review articles, conference abstracts or posters, hypothesis articles, or clinical trial registrations without published or publicly available results.

Study Selection and Data Extraction

All records retrieved from the three databases were imported into Rayyan for study management [24]. Duplicate records were deleted manually. Titles and abstracts of the remaining records were screened by two authors (YIZ and MM) independently. The potentially eligible studies underwent full-text screening.

Data were independently extracted into a standardized form by the same two authors. This form contained information on the first author, year of publication, study design, sample size, age of patient, cancer type, cancer stage, radiotherapy or CCRT regimen, molecular hydrogen intervention, control intervention, duration of hydrogen therapy, and outcomes assessed. Any discrepancies were resolved through discussion and consultation with author AM.

Risk-of-Bias and Publication Bias Assessment

The risk-of-bias assessment was carried out by the same two authors independently. The Cochrane Risk of Bias 2 (RoB 2) tool was used to assess randomized controlled trials (RCTs) [25], whereas the Newcastle-Ottawa Scale (NOS) was used to evaluate the retrospective observational study [26]. Any disagreements were resolved through discussion and consultation with author AM.

Assessment of publication bias was not considered appropriate and was therefore not conducted due to the limited number of studies (n = 3) included in the meta-analysis. According to the Cochrane Handbook, formal assessment of publication bias is generally not recommended when fewer than 10 studies are included because the available methods have limited statistical power [27]. 

Data Synthesis and Statistical Analysis

Quantitative synthesis of studies reporting comparable outcomes was performed using Review Manager (RevMan) version 5.4 (The Cochrane Collaboration). Higgins' I² statistic was used to assess statistical heterogeneity. Although the I² value was 0%, only three studies were included in the meta-analysis, limiting the reliability of statistical heterogeneity estimates [28]. The included studies also differed in tumor type, molecular hydrogen administration method, and study design, indicating the presence of methodological and clinical heterogeneity. Thus, a random-effects model was applied in the meta-analysis.

Speaking of dichotomous outcomes, effect estimates were calculated as risk ratios (RRs) and 95% confidence intervals (CIs) with the Mantel-Haenszel method. Sensitivity analyses were performed by restricting the analysis to RCTs and using a leave-one-out approach. Outcomes that could not be quantitatively synthesized because of differences in outcome measures or reporting methods were summarized narratively.

Results

Study Selection

A total of 4,754 records were identified from 3 electronic databases, including 874 from PubMed, 1,178 from Embase, and 2,702 from Web of Science. One additional record was identified from the reference lists of relevant reviews. After removal of 1,621 duplicate records, 3,133 records remained for title and abstract screening. After screening, 19 studies were defined as potentially eligible, and they underwent full-text eligibility assessment. Thirteen studies were excluded for the following reasons: conference abstracts or conference posters (n = 1), clinical trial registrations without published or publicly available results (n = 4), review articles (n = 3), animal studies (n = 3), and hypothesis articles (n = 2). Excluded studies were listed in the Appendices. Finally, seven studies were included in the qualitative synthesis [29-35], of which three were included in the quantitative synthesis [30-32]. The process of how studies were selected is illustrated in Figure 1.

**Figure 1:**
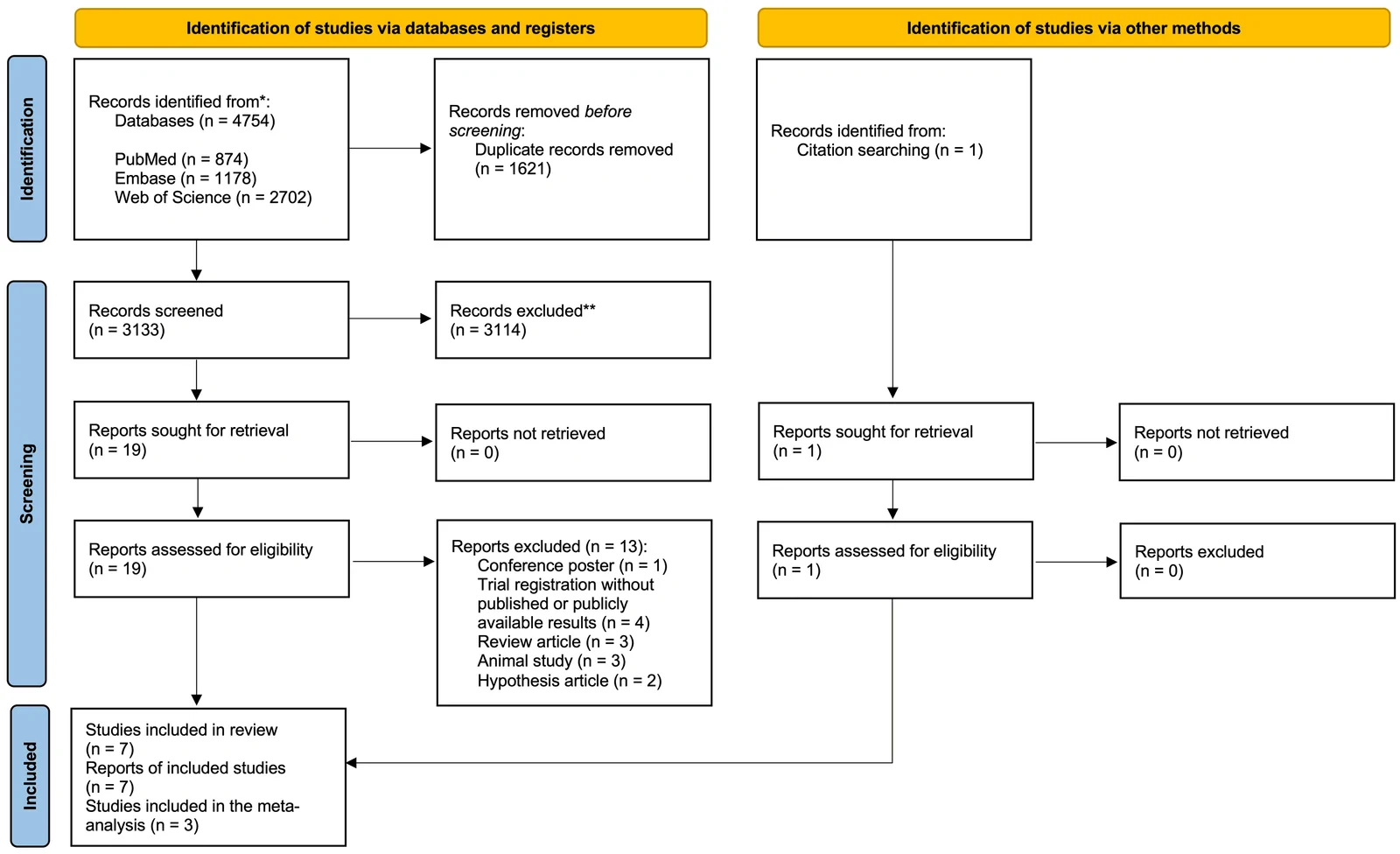
PRISMA 2020 flow diagram of study selection

Characteristics of Included Studies

Seven studies involving 222 patients were synthesized qualitatively, of which 3 studies involving 130 patients were synthesized quantitatively. The included studies comprised three RCTs, one retrospective observational study, two prospective single-arm studies, and one case report. These studies were published between 2011 and 2026. Patients received radiotherapy or CCRT for a range of malignancies, including head and neck, cervical, liver, and nasopharyngeal cancers. Molecular hydrogen was administered either by inhalation or in the form of hydrogen-rich water for drinking or gargling. Characteristics of the seven studies are summarized in Table 2.

**Table 2:** Characteristics of included studies CCRT: concurrent chemoradiotherapy, IMRT: intensity-modulated radiotherapy, H₂: molecular hydrogen, NLR: neutrophil-to-lymphocyte ratio, CRP: C-reactive protein, IL-6: Interleukin-6, PLR: platelet-to-lymphocyte ratio, dROMs: derivatives of reactive oxidative metabolites, BAP: biological antioxidant power.

Study (Author, year)	Study design	Sample size (I/C)	Age (yr)	Cancer type & Stage	(Chemo)Radiotherapy regimen	Molecular hydrogen intervention & duration	Control	Outcomes measured
Tsai et al. (2026) [29]	RCT	17 / 17, (16 / 15 analysed)	56.20 ± 10.15	Head and neck cancer stages 1-4	Radiotherapy or CCRT: 60 to 70 Gy (+ cisplatin)	Hydrogen water gargling (2.79 to 4.89 × 10^8 hydrogen bubbles/mL). Each participant got 20 mL, gargled for 30–60 seconds every 4 hours, at least 3 times per day. Duration: 2 weeks	Distilled water gargling	Oral mucositis; oral frailty; pain severity; quality of life
Liu et al. (2025) [30]	RCT	66 enrolled, 28 / 30 analysed	18 to 75	cervical cancer FIGO2018 stages II and III	CCRT: 45 to 50 Gy in 25 fractions over 5 weeks, or 6 Gy per fraction, administered in 5 sessions. (Additional 10 to 15 Gy if positive lymph nodes exist) + taxanes and platinum-based agents	On the same day as RT, inhalation of a hydrogen (66.6%)-oxygen (33.3%) gas mixture for 2 hours, 3 L/min. Duration: 25 days	No additional gas inhalation	Measures of hemoglobin, neutrophil, platelet, lymphocyte, CRP, NLR, PLR, IL-6; faecal occult blood positivity; radiation-induced intestinal toxicity; nutritional status; overall clinical enteritis severity; tumor response
Kang et al. (2011) [31]	RCT	25 / 24	21 to 82 (mean: 58.6)	Liver tumor stage not reported	Radiotherapy: 5040-6500 cGy for 7-8 weeks with a 6 MV system	Drink hydrogen-rich water (0.55~0.65 mM). Participants were instructed to drink 100-300 mL more than 10 times per day. Total consumption: 1500 mL-2000 mL per day. Duration: 6 weeks	Drink placebo water	Quality of life; oxidative stress biomarker: dROMs, BAP; liver function biomarker: aspartate aminotransferase, alanine aminotransferase, gamma-glutamyl transpeptidase, and total cholesterol; blood composition: counts of leukocytes, erythrocytes, and thrombocytes; tumor response
Hirano et al. (2021) [32]	Retrospective observational study	18 / 8, (16 / 7 analysed)	I: 65.9 ± 3.5 C: 54.6 ± 8.3	Tumor origin: ovary, stomach, colon, lung, prostate, breast, ureter, liver, bile duct, pancreas, and urinary bladder; TNM stage IV	IMRT: 5 to 20 minutes of Tomo therapy once per day for 1 to 4 weeks. The number of radiation times (I/C): 11.2 ± 1.1 / 10.7 ± 1.3 Total exposure dose (I/C): 410.2 ± 124.4 × 10^3 cm^3·Gy / 446.0 ± 101.8 × 10^3 cm^3·Gy	After each session of IMRT, 5% hydrogen gas inhalation for 30 minutes, 4 L/min in a health care chamber. Duration: 1 to 4 weeks	27% oxygen gas inhalation under 1.35 atm	Bone marrow damage evaluation: counts of leukocytes, erythrocytes, and thrombocytes; levels of hemoglobin and hematocrit; tumor response
Chitapanarux et al. (2024) [33]	Prospective single-arm pilot feasibility study	10	30–68 median: 58	locally advanced head and neck cancer (LAHNC) AJCC 8th edition: III–IVB	CCRT: 69.96 Gy, or 59.4 Gy, or 54 Gy to areas with different risks in 33 fractions. + weekly cisplatin or weekly carboplatin	Hydrogen gas inhalation (66% in air), 1.2 L/min, 1 hour per day, 1–2 hours before radiotherapy. Duration: median 49 days (range 47–63)	No control group	Feasibility of H₂ inhalation; safety of H₂ inhalation; acute toxicities during CCRT; tumor response
Chen et al. (2019) [34]	Case report	3	Case 1: 63; Case 2: 64; Case 3: 63	Nasopharyngeal cancer stage I or II	Radiotherapy: case 1: 72 Gy given in 36 fractions; case 2: 70 Gy in 36 fractions; case 3: 72 Gy in 40 fractions	Hydrogen gas inhalation (67% hydrogen and 33% oxygen), 3 L/min, 4 h each day. Duration: 1–2 months	No control group	Hearing threshold; middle ear function; ear examination; patient-reported hearing improvement; hearing-aid use
Kong et al. (2022) [35]	Prospective single-arm interventional study	34 (17 analyzed)	47 to 67 (median: 58)	Nasopharyngeal carcinoma; AJCC 7th edition: Stage I-IV	Radiotherapy or CCRT: 68–72 Gy, 70.0 Gy, and 50.0–60.0 Gy to different areas. 34 to 38 fractions in total, 1.8–2.0 Gy per fraction, once a day, five times a week. (+ cisplatin, intravenous drips, once per week.)	Hydrogen (2.0 L/min)-oxygen (1.0 L/min) mixture of gas inhalation, 3–6 h per day. Duration: 4–12 weeks	No control group	Eustachian tube dysfunction; hearing threshold; tympanic membrane (otoscopy) score; tympanogram score; safety / adverse events

Risk-of-Bias Assessment

Risk of bias was assessed based on study design for the three studies included in the meta-analysis [30-32]. The two RCTs were evaluated using the RoB 2 tool across five domains (D1-D5). Overall, Liu et al. (2025) was judged to be at high risk of bias, primarily due to a high risk in D2 (deviations from the intended interventions). Kang et al. (2011) was judged to have some concerns overall because of concerns in D1 (randomization process) and D5 (selection of the reported result) (Figure 2). The retrospective observational study by Hirano et al. (2021) was assessed using the NOS for cohort studies and received eight out of nine stars, indicating good methodological quality (Figure 3).

**Figure 2:**
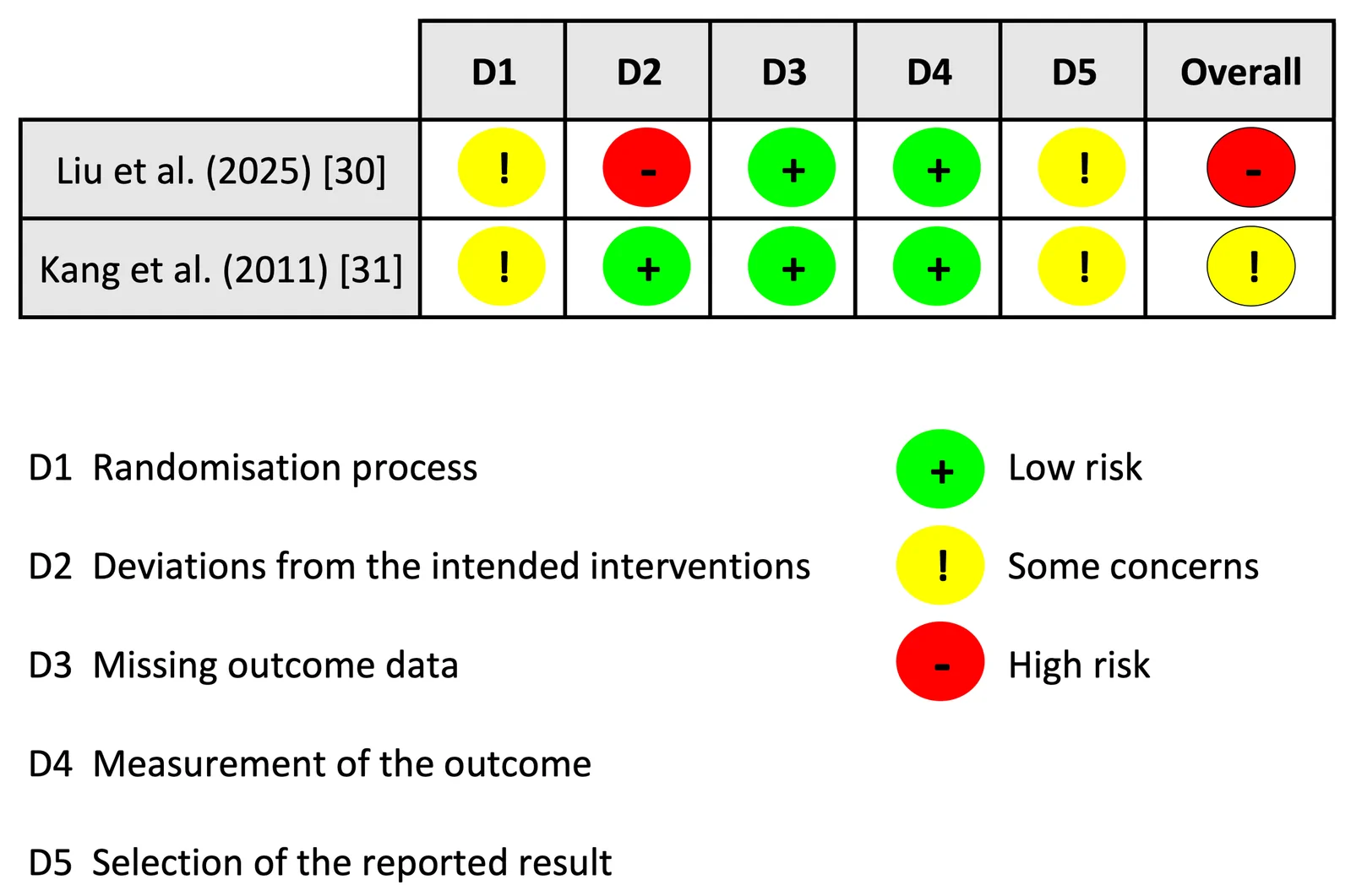
Risk-of-bias assessment using the RoB2 tool

**Figure 3:**
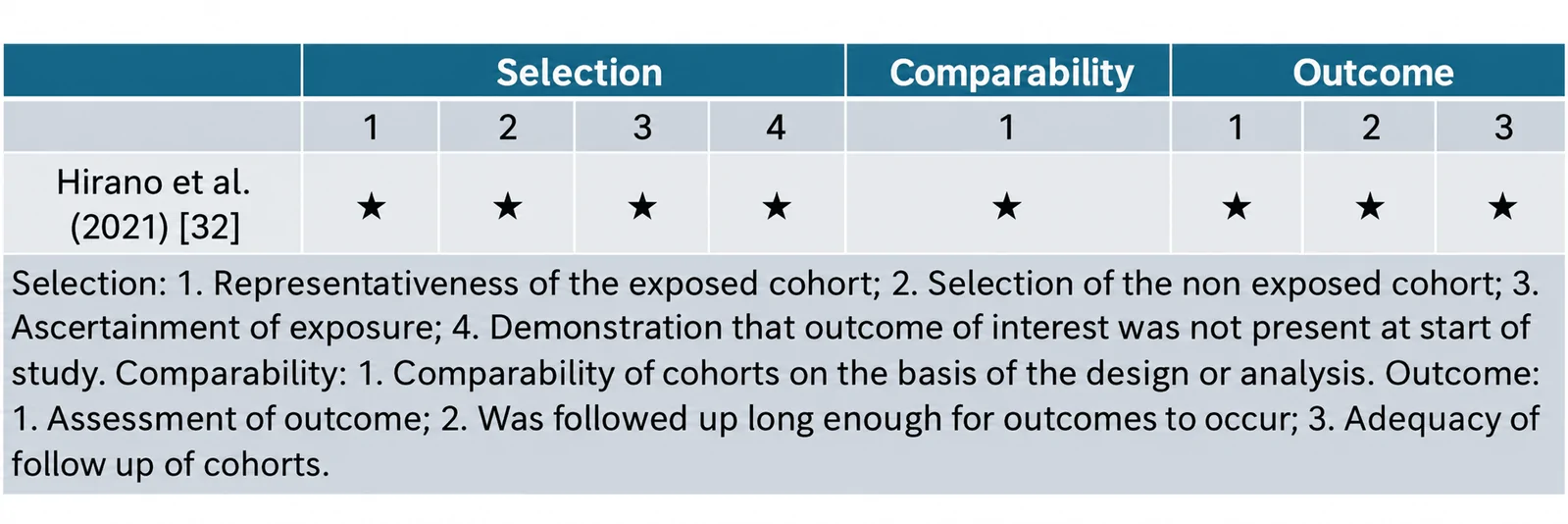
Risk-of-bias assessment using the NOS NOS: Newcastle-Ottawa Scale

Quantitative Synthesis (Meta-Analysis)

Tumor response was assessed using the original or modified Response Evaluation Criteria in Solid Tumours (RECIST) across the included studies. Tumor response was categorized as complete response (CR), partial response (PR), stable disease (SD), or progressive disease (PD). Patients with CR or PR were considered responders (i.e., patients contributing to the objective response rate), whereas those with SD or PD were considered non-responders. In the forest plot, events represent responders. The effect of molecular hydrogen on tumor response was quantified using the risk ratio (RR) (Figure 4).

**Figure 4:**
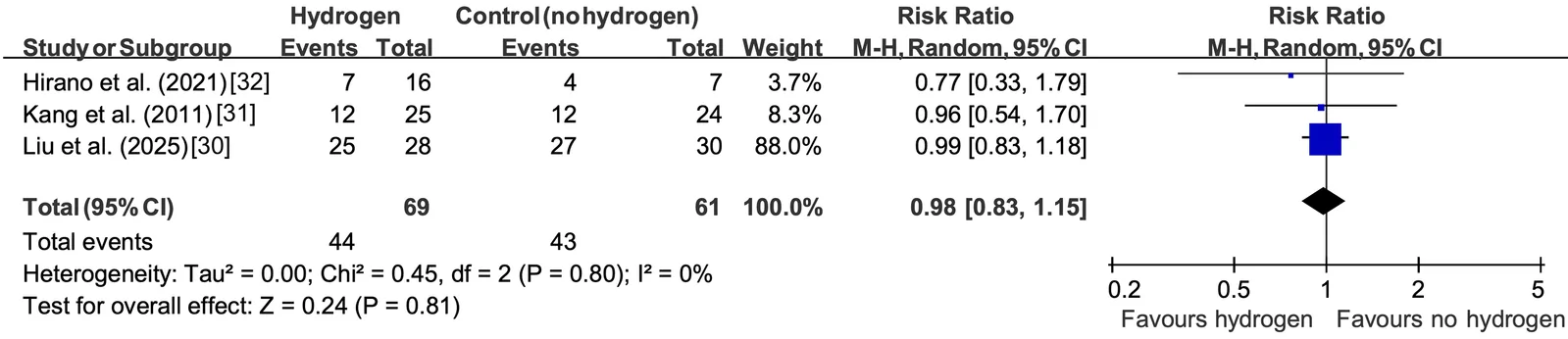
Forest plot of the effect of molecular hydrogen on tumor response in cancer patients receiving radiotherapy or CCRT CCRT: concurrent chemoradiotherapy

In each individual study, the 95% CI for the RR included 1, indicating no statistically significant difference in tumor response between the hydrogen and control groups. The pooled analysis likewise showed no significant difference in tumor response between these two groups (RR = 0.98, 95% CI 0.83-1.15, P = 0.81). However, due to the limited sample size and the relatively wide 95% CI, there may be substantial uncertainty around the pooled estimate.

Sensitivity Analyses

The pooled estimate was also calculated using only the two RCTs. The result (RR = 0.99, 95% CI: 0.84-1.17) was broadly consistent with the primary analysis in Figure 4, indicating that inclusion of the retrospective observational study did not materially influence the overall effect estimate (Figure 5).

**Figure 5:**
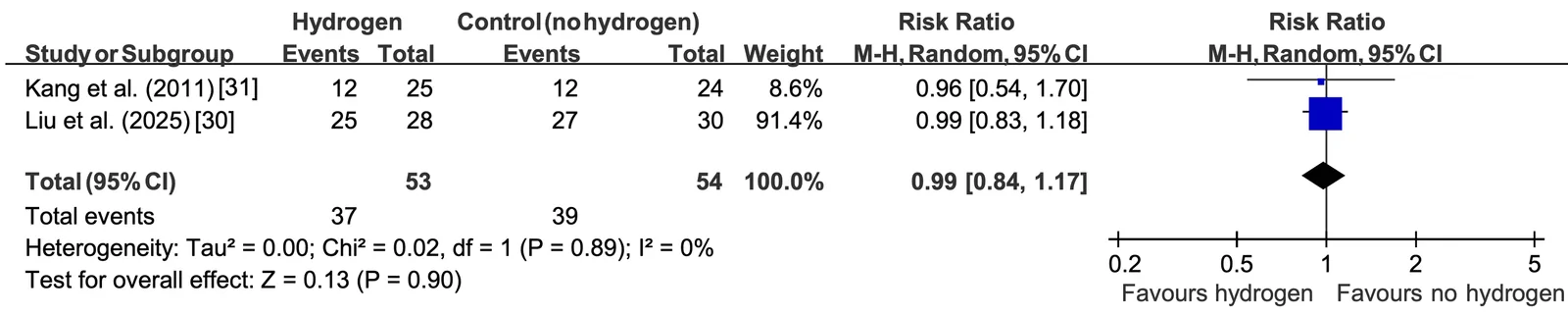
Sensitivity analysis restricted to two RCTs

A leave-one-out method was used for sensitivity analysis to assess how robust the pooled estimate for tumor response was (Table 3). Sequential exclusion of each study did not materially alter the RR compared with the primary analysis. None of the pooled estimates reached statistical significance (all overall P ≥ 0.05), indicating that the conclusion remained unchanged regardless of which study was excluded. Although exclusion of Liu et al. (2025) [30] also maintained the non-significant result, the 95% CI substantially widened, indicating increased uncertainty around the pooled estimate.

**Table 3:** Sensitivity analysis of the pooled risk ratio for tumor response

Analysis	RR (95% CI)	Overall P	I²
Primary analysis	0.98 (0.83, 1.15)	0.81	0%
Excluding Hirano et al. (2021) [32]	0.99 (0.84, 1.17)	0.9	0%
Excluding Kang et al. (2011) [31]	0.98 (0.83, 1.17)	0.83	0%
Excluding Liu et al. (2025) [30]	0.89 (0.56, 1.44)	0.65	0%

Radiation-Induced Toxicities

Several included studies reported that molecular hydrogen was associated with alleviation of radiation-induced toxicities in cancer patients receiving radiotherapy or CCRT. In patients with head and neck cancer, hydrogen-rich water gargling reduced the severity of treatment-induced oral mucositis and oral pain [29]. In patients with cervical cancer receiving CCRT, hydrogen gas inhalation preserved gastrointestinal mucosal integrity and attenuated acute radiation enteritis [30]. In patients with liver cancer receiving radiotherapy, consumption of hydrogen-rich water reduced oxidative stress and maintained biological antioxidant activity throughout treatment [31]. In cancer patients receiving intensity-modulated radiotherapy, hydrogen gas inhalation protected against radiation-induced bone marrow damage [32]. Two studies involving patients with nasopharyngeal carcinoma reported that hydrogen gas inhalation ameliorated radiotherapy- or CCRT-induced hearing impairment, primarily through improvements in hearing thresholds [34,35]. The feasibility and safety of hydrogen gas inhalation were demonstrated in a pilot clinical study of patients with locally advanced head and neck cancer receiving CCRT. All patients completed the planned hydrogen therapy regimen, and no hydrogen-related adverse events were reported throughout the treatment period [33].

Quality of Life 

Quality of life (QOL) was assessed in two studies [29,31]. Both studies reported improvements in QOL following molecular hydrogen therapy in cancer patients receiving radiotherapy or CCRT, although different questionnaires developed by the European Organisation for Research and Treatment of Cancer (EORTC) were used, namely, the EORTC QLQ-H&N35 and a modified version of the EORTC QLQ-C30 [36]. In patients with head and neck cancer, hydrogen-rich water was administered for two weeks after completion of radiotherapy [29]. In patients with liver cancer, hydrogen-rich water was consumed during six weeks of a 7-8-week radiotherapy course [31].

Hematological Outcomes

Hematological outcomes were reported in three studies [30-32]. Two of them reported that molecular hydrogen administration did not significantly alter hematological parameters compared with the control group, suggesting that hydrogen did not adversely affect hematological safety [30,31]. In contrast, Hirano and colleagues reported that hydrogen inhalation significantly attenuated radiation-induced reductions in leukocyte and thrombocyte counts, while erythrocyte count, hemoglobin concentration, and hematocrit remained unchanged [32]. Thus, these studies suggest that molecular hydrogen does not adversely affect hematological parameters and may attenuate radiation-induced bone marrow suppression.

Discussion

This systematic review and meta-analysis found no evidence of reduced tumor response associated with molecular hydrogen in patients receiving radiotherapy or concurrent chemoradiotherapy. The included studies suggested that molecular hydrogen may alleviate a broad range of radiation-induced toxicities. Molecular hydrogen therapy was also associated with improvements in quality of life and did not adversely affect hematological parameters, supporting its favourable safety profile.

Consistent with previous reviews, which suggested that molecular hydrogen may provide radioprotection without compromising tumor response [6-8], the present meta-analysis provides a quantitative synthesis of the available clinical evidence, with no significant difference observed in tumor response between the hydrogen and control groups. The selective radioprotective effects of molecular hydrogen may be partly explained by the distinct redox status of normal and cancer cells. Compared with normal cells, cancer cells generally produce higher levels of ROS and exhibit elevated basal oxidative stress because of metabolic reprogramming. Consequently, cancer cells are more vulnerable to further increases in oxidative stress induced by radiotherapy [37-40]. Although molecular hydrogen selectively scavenges •OH, water radiolysis also generates other ROS, including hydrogen peroxide (H₂O₂) [9]. These ROS may further increase oxidative stress beyond the tolerance threshold of cancer cells, thereby promoting cancer cell death despite molecular hydrogen administration [41,42].

In addition to its radioprotective properties, molecular hydrogen has also been reported to exert antitumor effects. Molecular hydrogen can remodel the tumor microenvironment, thereby enhancing systemic antitumor immunity [43]. Molecular hydrogen has also been shown to inhibit cancer growth through different signalling pathways [44,45]. These preclinical findings provide potential biological support for the observation that molecular hydrogen was not associated with reduced tumor response in the included clinical studies.

The potential radioprotective effects of molecular hydrogen may involve its antioxidant, anti-inflammatory, anti-apoptotic, and gene-regulatory properties in normal tissues, thereby potentially improving the quality of life of cancer patients [7].

Several limitations of this review should be acknowledged. First, only 3 studies involving 130 patients were available for quantitative synthesis, limiting the statistical power of the meta-analysis. Second, the included studies were clinically and methodologically heterogeneous with respect to cancer type, radiotherapy regimen, molecular hydrogen administration method, and treatment duration. Moreover, subgroup analyses could not be performed because only three studies were available for meta-analysis, making it impossible to explore whether these differences influenced the pooled estimate. Third, one included study was a retrospective observational study, and one RCT had a high risk of bias. These factors reduce confidence in the pooled estimate and lower the overall certainty of the evidence. Fourth, the included studies were restricted to those published in English. Given that much of the research on molecular hydrogen has been conducted in Japan, China, and Korea, the exclusion of studies published in other languages represents a potential limitation. Finally, secondary outcomes were assessed using different outcome measures across studies, limiting the analysis to a narrative synthesis. Therefore, large, well-designed, multicentre RCTs with standardized study protocols and outcome measures are needed to further validate the findings of this meta-analysis.

## Conclusions

Overall, this systematic review and meta-analysis suggest that molecular hydrogen may have radioprotective effects in cancer patients receiving radiotherapy or concurrent chemoradiotherapy, with the currently available evidence showing no significant reduction in tumor response. However, the small number of studies and patients precludes definitive conclusions regarding preservation of antitumor efficacy.

## Appendices

List of 13 records excluded during full-text screening:

1. Chitapanarux I, Onchan W, Chakrabandhu S, Muangwong P, Autsavapromporn N, Ariyanon T: TCTR20230627002: Feasibility of hydrogen gas inhalation in chemoradiation for head and neck cancer. Radiother Oncol. 2024, 197:S174-5. 10.1016/S0167-8140(24)04074-X

2. Kaohsiung Medical University Chung-Ho Memorial Hospital: The Effect of Hydrogen Water in Oral Mucositis, Oral Frailty, Pain and Quality of Life in Head and Neck Cancer Patients After Therapy. clinicaltrials.gov; 2023. 

3. Kaohsiung Medical University Chung-Ho Memorial Hospital: Effectiveness Verification of Using Hydrogen Inhalation to Improve Fatigue, Pain and Quality of Life in Cancer Patients. clinicaltrials.gov; 2023. 

4. Moran C: Hydrogen Rich Water to Mitigate Acute Mucositis During Radiation Therapy. clinicaltrials.gov; 2025. 

5. Abuawad Z: Effectiveness of Vitamin E and Molecular Hydrogen-Rich Water on Radiation Therapy-Induced Adverse In Adult Patients With Rectal Carcinoma in Al-Quds, Palestine. clinicaltrials.gov; 2021. 

6. Au TY, Darwiche F, Benjamin S, Assavarittirong C: Hydrogen inhalation: a potential treatment for radiotherapy/chemotherapy-induced hearing loss in cancer patients. Med Gas Res. 2026, 16:86-7. 10.4103/mgr.MEDGASRES-D-25-00053

7. Terasaki Y, Suzuki T, Ohsawa I, et al.: Hydrogen-Supplemented Drinking Water Protects Against Naphthalene-Gefitinib Induced Lung Injury from Inflammation-Associated Oxidative Stress. Am J Respir Crit Care Med. 2014, 189:A3487-A3487. 10.1164/ajrccm-conference.2014.189.1_MeetingAbstracts.A3487

8. Chuai Y, Zhao L, Ni J, et al.: A possible prevention strategy of radiation pneumonitis: Combine radiotherapy with aerosol inhalation of hydrogen-rich solution. Med Sci Monit. 2011, 17:HY1-4. 10.12659/MSM.881698

9. Chuai Y, Qian L, Sun X, Cai J: Molecular hydrogen and radiation protection. Free Radic Res. 2012, 46:1061-7. 10.3109/10715762.2012.689429

10. Xiao H, Li Y, Luo D, et al.: Hydrogen-water ameliorates radiation-induced gastrointestinal toxicity via MyD88’s effects on the gut microbiota. Exp Mol Med. 2018, 50:e433-e433. 10.1038/emm.2017.246

11. Xu K, Sun G, Wang Y, et al.: Mitigating radiation-induced brain injury via NLRP3/NLRC4/Caspase-1 pyroptosis pathway: Efficacy of memantine and hydrogen-rich water. Biomed Pharmacother. 2024, 177:116978. 10.1016/j.biopha.2024.116978

12. Hirano S, Ichikawa Y, Sato B, Yamamoto H, Takefuji Y, Satoh F: Molecular Hydrogen as a Potential Clinically Applicable Radioprotective Agent. Int J Mol Sci. 2021, 22:4566. 10.3390/ijms22094566

13. Chen Y, Zong C, Guo Y, Tian L: Hydrogen-rich saline may be an effective and specific novel treatment for osteoradionecrosis of the jaw. Ther Clin Risk Manag. 2015, 11:1581-1585. 10.2147/TCRM.S90770
